# How Do “Mute” Cicadas Produce Their Calling Songs?

**DOI:** 10.1371/journal.pone.0118554

**Published:** 2015-02-25

**Authors:** Changqing Luo, Cong Wei, Christian Nansen

**Affiliations:** 1 Key Laboratory of Plant Protection Resources and Pest Management, Ministry of Education, Entomological Museum, Northwest A&F University, Yangling, Shaanxi 712100, China; 2 Department of Entomology and Nematology, UC Davis Briggs Hall, Room 367, University of California Davis, Davis, California, United States of America; University of Brasilia, BRAZIL

## Abstract

Insects have evolved a variety of structures and mechanisms to produce sounds, which are used for communication both within and between species. Among acoustic insects, cicada males are particularly known for their loud and diverse sounds which function importantly in communication. The main method of sound production in cicadas is the tymbal mechanism, and a relative small number of cicada species possess both tymbal and stridulatory organs. However, cicadas of the genus *Karenia* do not have any specialized sound-producing structures, so they are referred to as “mute”. This denomination is quite misleading, as they indeed produce sounds. Here, we investigate the sound-producing mechanism and acoustic communication of the “mute” cicada, *Karenia caelatata*, and discover a new sound-production mechanism for cicadas: i.e., *K. caelatata* produces impact sounds by banging the forewing costa against the operculum. The temporal, frequency and amplitude characteristics of the impact sounds are described. Morphological studies and reflectance-based analyses reveal that the structures involved in sound production of *K. caelatata* (i.e., forewing, operculum, cruciform elevation, and wing-holding groove on scutellum) are all morphologically modified. Acoustic playback experiments and behavioral observations suggest that the impact sounds of *K. caelatata* are used in intraspecific communication and function as calling songs. The new sound-production mechanism expands our knowledge on the diversity of acoustic signaling behavior in cicadas and further underscores the need for more bioacoustic studies on cicadas which lack tymbal mechanism.

## Introduction

The use of sounds in communication, both within and between species, occurs widely among vertebrates and arthropods. Recently, there has also been a growing interest and research into the possible role of sound communication in the Plant Kingdom [1,2]. Among animals, sound-based communication is used in a variety of behavioral contexts, such as, mating behavior and territorial defense [3,4].

Insects have evolved a marked diversity of methods to produce sounds involving almost any part of the insect’s exoskeleton [5], and the five main sound-producing mechanisms are: 1) vibration, 2) percussion, 3) stridulation, 4) click mechanism, and 5) air expulsion [6–8]. Among insects, cicadas (Hemiptera, Cicadoidea) are among the best-studied groups in terms of sound production [9]. The most common structure used by cicadas to produce sounds is the tymbal organ which is essentially composed of a ribbed membrane at the base of the abdomen and an attached muscle. The sound is generated when the tymbal muscle activity deforms the stiff membrane [10–12]. In addition, different types of stridulatory organs have been described in a relatively small number of cicada species [13]. The stridulatory organ found in these cicada species, similar to that observed in crickets, katydids and grasshoppers, consists of a scraper (usually a part of one of the tegminal veins) and a file (a specialized part of either the mesonotum, or the pronotum, or the hind wing) [14,15].

Remarkably, the “mute” cicadas of the genus *Karenia* Distant (Cicadidae, Cicadettinae) have neither tymbals nor stridulatory organs [16]. This genus comprises only five species distributed in China, Burma and Vietnam [17]. Although lacking any specialized sound-producing structures, these cicadas can produce sounds using their forewings [16]. Recently, Wei et al. [18] reported sound production in *K*. *chama* and discovered that this species exhibited an atypical behavior, i.e., the male adults can be easily attracted to sounds produced by clapping of hands, knocking of bamboo sticks, breaking of twigs, and chopping of wood. However, the detailed sound-producing mechanism and the morphological structures responsible for sound production in the *Karenia* have not been investigated. Furthermore, acoustic properties of the sounds produced by these cicadas are also unclear.

There is growing interest in the use of high spatial and spectral resolution reflectance profiling, such as hyperspectral imaging, as part of providing quantitative evidence of difference between objects that appear very similar or identical to the human eye. For instance, analyses of reflectance profiles have been used to accurately classify dry field peas with/without internal infestation by a weevil [19]. The noteworthy aspect of this study was that 12 different field peas varieties were included in the study, and they ranged considerably in colours. However, despite a marked variation in colours, there was a consistent and detectable response to pea weevil infestation. Nansen et al. [20] demonstrated that three species of minute juvenile egg-parasitoids (*Trichogramma*) developing inside moth host eggs could be accurately classified based on the reflectance profiles acquired from the host eggs. Similarly, there are studies of how reflectance profiling has been used in taxonomic studies of fossil insects [21]. Finally, there are numerous examples of studies in which reflectance profiling has been used in classification of insects, including species of stored grain insects [22], tobacco budworm [(*Heliothis virescens* (F.) (Lep.: Noctuidae)] and corn earworm [(*Helicoverpa zea* (Boddie) (Lep.: Noctuidae)] [23], and to age-grade biting midges [*Culicoides sonorensis* Wirth & Jones (Dip.: Ceratopogonidae)] [24]. Klarica et al. [25] used imaging spectroscopy to discriminate cryptic species of ants [*Tetramorium caespitum* (L.) and *T*. *impurum* (Foerster) (Hym.: Formicidae)]. Based on this extensive and growing use of reflectance-based analyses in behavioral and taxonomic studies of insects and other animals, we apply this technology to the detailed analyses of sound-producing structures of *Karenia* cicadas.

In the present study, we provide the first comprehensive analysis of sound-producing behavior and the morphology of structures involved in sound production by males of *Karenia caelatata*. The specific objectives were to: (1) analyse the mechanism of sound production; (2) provide a description of the acoustic properties of the sounds produced by *K*. *caelatata* males; and (3) explore whether the sounds produced by males play a role in intraspecific communication.

## Materials and Methods

### Ethics Statement

No specific permits were required for the cicada species *Karenia caelatata* used for this study in China, because this cicada species is not privately-owned or protected in any way. The cicada *K*. *caelatata* was not included in the “List of Protected Animals in China”. This study did not involve endangered or protected species.

### The study site and species

Field investigations were performed during the summer emergence of *Karenia caelatata* (July–August) in 2012 and 2013 at the Forestry Station Huoditang, Ningshan County, Shaanxi Province, China, which is located in the Qinling Mountains (33°18′N, 108°20′E). This population occurs at elevations between 1500 and 2000 m, and feeds mainly on *Quercus aliena* var. *acuteserrata*.

### Behavioral observations and sound analysis

Sound production behavior exhibited by *K*. *caelatata* males was observed both under natural conditions and in cages. Males of *K*. *caelatata* were collected by light trapping. Captured males were kept in cages (1.0×1.0×1.0 m; netted with white nylon) and fed freshly cut branches of *Q*. *aliena* var. *acuteserrata*. Captured males were observed individually in the cage, and observations were performed within two days after capture. The sound production activity was video-recorded using a Nikon Coolpix P100 digital camera (Nikon Corporation, Indonesia). Effort was made to minimize the disturbance to the cicadas during behavioral observation by reducing the noise and avoiding sudden movements of the observer.

The sounds produced by *K*. *caelatata* males were recorded using a linear PCM recorder with stereo microphones (PCM-D50, Sony, China; frequency range 20–20000 Hz and a 44.1 kHz/16 bit sampling resolution). The sounds were recorded in WAV file format, and stereo recordings were converted to mono at a sampling rate of 44.1 kHz and resolution of 16 bits. Acoustic analysis was conducted using the Raven Pro 1.4 (The Cornell Lab of Ornithology, Ithaca, NY, USA) and the Seewave package [26], a custom-made library of the R software platform [27]. Terminology for the description of acoustic signals follows that of Alexander [28].

The recordings and behavioral observations were made between 10:00 am and 2:00 pm, as a priori observations had shown this to be the time period with highest acoustic activity by the male cicadas. The ambient temperature ranged from 29 to 34°C.

### Morphological observation and measurements

We examined the morphology of the sound-producing structures (including forewing, operculum, cruciform elevation, and wing-holding groove on the scutellum) with an Olympus SZX 10 stereomicroscope (Olympus Corporation, Tokyo, Japan). Micrographs were captured with a Retiga 2000R digital camera (QImaging, Canada) mounted on a Nikon SMZ 1500 stereoscopic zoom microscope (Nikon Corporation, Tokyo, Japan), and then 80 sequential shots at different focal depths were processed using the Auto-Montage Pro software to generate a single composite image.

We compared the relative forewing length (the ratio of forewing length to body length) of *K*. *caelatata* males with that of 11 other representative cicada species (including another mute cicada species *K*. *chama*), from the three subfamilies of Cicadidae (i.e., Tettigadinae, Cicadettinae, and Cicadinae), which have different sound-producing mechanisms (for details see Table 1). Body length (from tip of head to tip of abdomen) and forewing length (distance from base of right forewing articulation to tip of wing) were measured to the nearest 0.01 mm using a vernier caliper. Twenty-five male specimens per species were used for measurement, except that only nine individuals of the cicada *K*. *chama* were available for measurement.

**Table 1:** Twelve cicada representatives of the three subfamilies of Cicadidae investigated in this study.

Species	Code	Tymbal organs	Stidulatory organs	Tribe	Subfamily
*Karenia caelatata*	Cae	Without	Without	Sinosenini	Cicadettinae
*Karenia chama*	Cha	Without	Without	Sinosenini	Cicadettinae
*Katoa tenmokuensis*	Kat	With	Without	Taphurini	Cicadettinae
*Cicadetta shansiensis*	Cic	With	Without	Cicadettini	Cicadettinae
*Kosemia yezoensis*	Lep	With	Without	Cicadettini	Cicadettinae
*Huechys sanguinea*	Hue	With	Without	Huechysini	Cicadettinae
*Pomponia linearis*	Pom	With	Without	Cicadini	Cicadinae
*Cryptotympana atrata*	Cry	With	Without	Cryptotympanini	Cicadinae
*Platypleura kaempferi*	Pla	With	Without	Platypleurini	Cicadinae
*Meimuna mongolica*	Mei	With	Without	Dundubiini	Cicadinae
*Platylomia bocki*	Boc	With	Without	Dundubiini	Cicadinae
*Subpsaltria yangi*	Sub	With	With	Tibicinini	Tettigadinae

### Costa-ablation experiments

**Fig 1:**
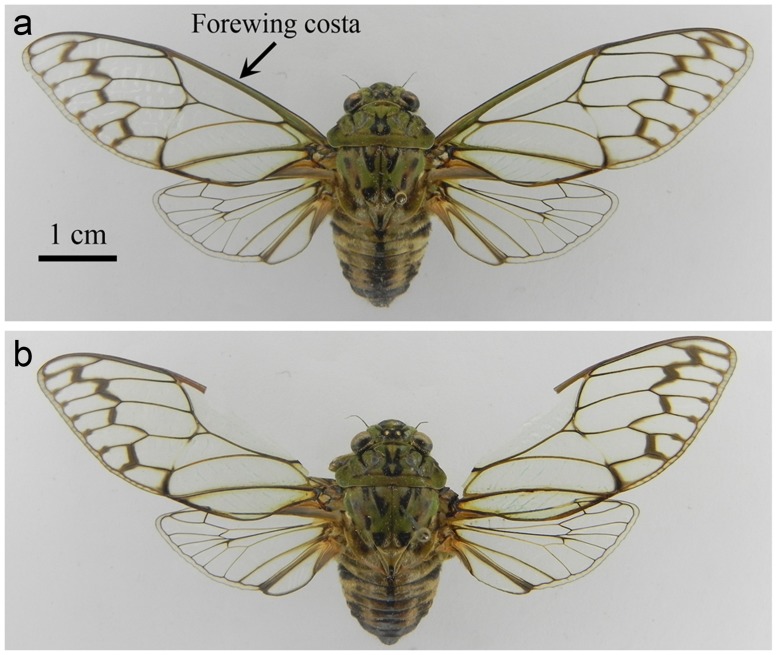
Costa-ablation experiment. (a) A male with wings spreaded to show the forewing costa. (b) A male with wings spreaded to show the forewing costa removed.

Behavioral observations indicated that the costa of forewing might play a crucial role in sound production of *K*. *caelatata* males. Costa-ablation experiment was conducted to determine experimentally whether the forewing costa was essential for sound production. A male was placed in a cage (1.0×1.0×1.0 m, netted with white nylon), and sound-producing behavior of the caged male was observed. Then, the costa of each forewing of the male was carefully removed with a surgical scissors (Fig. 1a, b), and sound-producing behavior exhibited by the male was observed again. The acoustic behavior of the male before and after ablation of the forewing costa was compared in detail. We ran this scraper-ablation experiment with a total of 30 males, and all experiments were carried out between 10:00 am and 2:00 pm.

### Reflectance-based analysis of forewing costa

**Fig 2:**
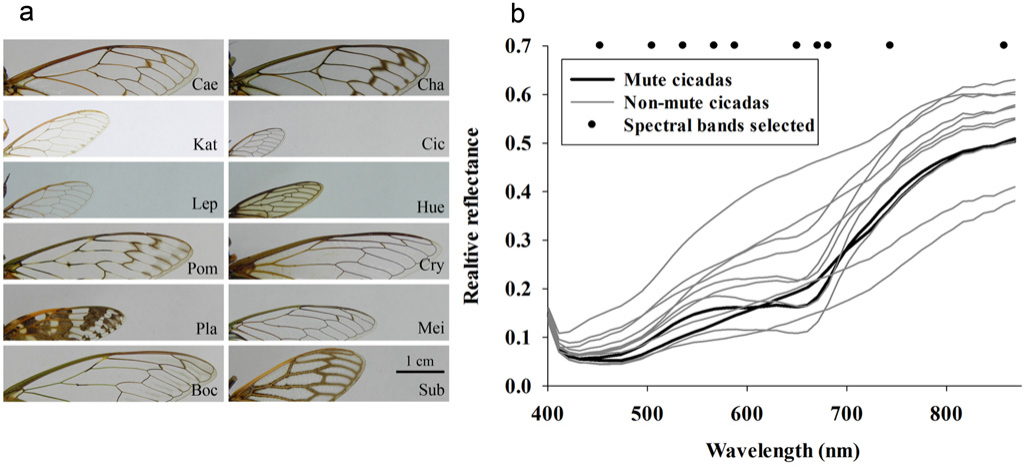
Forewing costas and average reflectance profiles of the 12 species included in this study. (a) Average reflectance profiles. Scale bar for all panels in (a) = 1 cm. (b) Average reflectance profiles. The dots in (b) denote the 23 location of the spectral bands selected for the classification of difference between non-mute and mute cicadas.

We used analysis of hyperspectral imaging data to quantify species-specific variation in forewing costa. That is, we predicted forewing costa would vary among species in terms of biochemical composition and physical structure, and this species-specific variation could be detected based on average reflectance profiles acquired from forewing costa. Consequently, we acquired hyperspectral imaging data from both forewing costas of five specimens of all cicada species in Table 1. Pixels representing forewing costa were carefully selected and averaged to obtain average reflectance profiles from individual forewing costa were generated (Fig. 2a). We used a hyperspectral spectral camera (PIKA II, Resonon Inc., Bozeman, MT) with the lens 13 cm above mounted cicadas, and reflectance data were acquired with the spatial resolution of 15 by 10 pixels per mm^2^. The main specifications of the spectral camera are as follows: interface, Firewire (IEEE 1394b); output, digital (12 bit); angular field of view of 7 degrees. The objective lens had a 35 mm focal length (maximum aperture of F1.4), optimized for the near-infrared and visible near-infrared spectra. Hyperspectral images were collected with artificial lighting from 15 W, 12 V LED light bulbs mounted on either side of the lens. A piece of white teflon (K-Mac Plastics, MI, USA) was used for white calibration, and “relative reflectance” was referred to proportional reflectance compared to reflectance obtained from teflon, and relative reflectance values ranged between 0 and 1. The original spectral data consisted of 240 spectral bands from 392–889 nm (spectral resolution = 2.1 nm). However prior to analysis, we omitted the first and last 5 spectral bands, as these spectral bands are associated with proportionally higher levels of stochasticity/noise. The remaining 230 spectral bands were “binned” (averaged) into 5-band intervals (increased the spectral resolution from 2.1 to 10.5 nm), which resulted in 46 spectral bands from 405–875 nm being included in the analysis. The spectral binning was conducted to increase classification accuracy [29] and to reduce the risk of model over-fitting [30–32].

### Acoustic playback experiments

The most common acoustic signals produced by male cicadas are calling songs. The calling songs can attract conspecific females and males in many cicada species [33–36]. To determine whether the sounds produced by *K*. *caelatata* males were used as calling songs, we conducted acoustic playback experiments in the field to test if males and females of the natural population could be attracted to the sounds.

A high-quality sound recording (i.e., having high amplitude relative to background noise and no overlap with other sounds) of one male of *K*. *caelatata* was selected to be used in playback experiments. Playbacks were conducted using a Sony PCM-D50 Linear PCM Recorder and a Mogic Q2 loudspeaker (frequency response, 150–20000 Hz). A digital sound level meter (GM1357, Benetech; fast response, A weighting) was used to measure sound pressure levels. The peak output intensity of the loudspeaker was adjusted to 65 dB SPL measured at 20 cm from the loudspeaker. The loudspeaker was placed on the ground among the vegetation. There was a 20-min control period in which no sound was presented, followed by a 20-min test period in which the acoustic stimulus was emitted by the loudspeaker. During the control and test periods, we recorded whether males and females of the natural population were attracted to the loudspeaker and the number of individuals attracted was counted, aiming to address the function of the acoustic signals emitted by *K*. *caelatata* males. The playback experiment was repeated 15 times, and all experiments were conducted between 10:00 am and 2:00 pm.

### Statistical analyses

All statistical analyses were performed with PC-SAS 9.3 (SAS Institute, NC). We used tukey analysis of variance (proc anova option = tukey) to compare body length and forewing measurements among the cicada species included in this study. Regarding analysis of the 120 average reflectance profiles (12 species × 5 specimens × 2 forewing costas) acquired from forewing costas, we used linear discriminant analysis [37]. Linear discriminant analysis has been widely used in analysis of reflectance profiles in which the objective is to compare and differentiate discrete classes of: tobacco leaves [38], food [39,40], seed [41], and feed [42]. In this study, we compared average reflectance profiles acquired from forewing costas among the 12 cicada species. Initially, we conducted a forward stepwise discriminant analysis (proc stepwise) to select the spectral bands (out of the 46 spectral bands), which contributed the most to the classification of forewing costas based on reflectance values in individual spectral bands. This data processing step led to selection of 10 spectral bands, which were used for further analysis. A random number function was used to divide the 120 average reflectance profiles so that 80% of the data was used as training data and the remaining 20% was used as independent validation. This random division of the 120 average reflectance profiles was repeated 5 times. For each of the 5 randomizations, a linear discriminant analysis (proc discrim) was performed, and the classification accuracy of linear discriminant scores generated on the basis of the training data set was assessed the classification sensitivity (ability to positively detect mute cicadas), and specificity (ability to positively detect non-mute cicadas) of the classification based on independent validation data.

## Results

### Sound-producing structures and mechanism

On the basis of behavioral observations of individuals when producing sounds, we conclude that the primary structures involved in sound production of *K*. *caelatata* males are the forewing, cruciform elevation, wing-holding groove on the scutellum, and the operculum.

**Fig 3:**
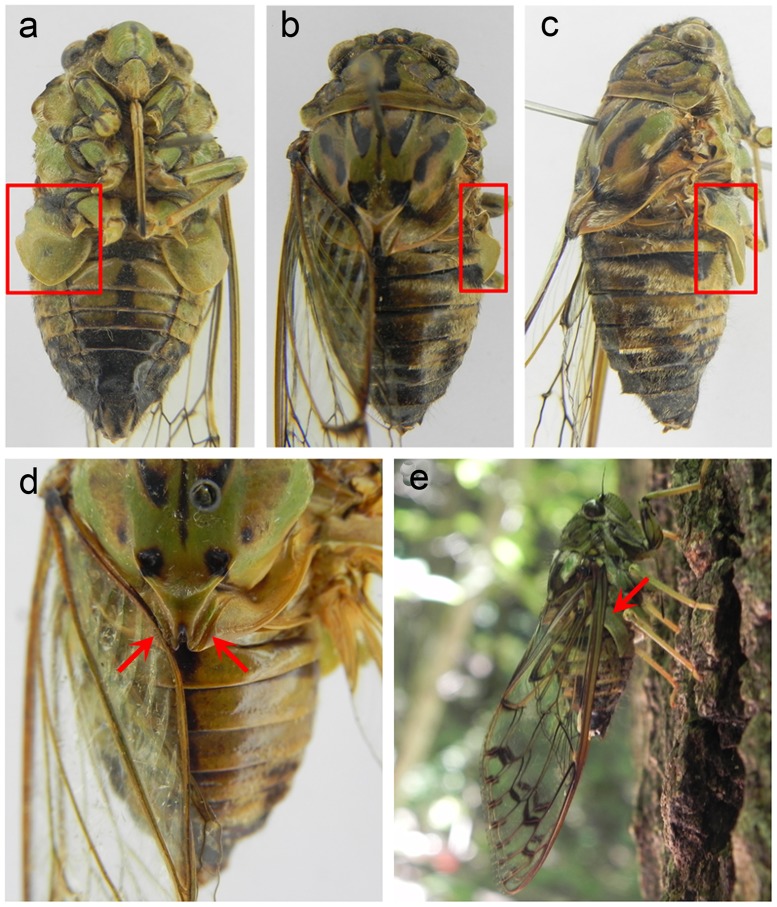
The sound-producing structures of *K*. *caelatata*. (a, b, c) Ventral, dorsal, and lateral view of a male with red box indicating the operculum involved in sound production. (d) Right red arrow marks the wing-holding groove of a male with right wing spread; left red arrow indicates that the basal posterior margin of the forewing is fixed in the wing-holding groove. (e) The forewing costa is supported over the operculum (red arrow).

The operculum of *K*. *caelatata* males, unlike that of species belonging to other cicada genera, was strongly modified in such a way that the base of the outer margin of the operculum was strongly upward curved and reached far beyond the lateral margin of the body (Fig. 3a–c). When the *K*. *caelatata* males were at rest, the forewings were held roof-like over the body; the basal inner margin of the forewing was locked in the wing-holding groove which was located much closer to the lateral margin of the V-shaped cruciform elevation than that in other cicadas (Fig. 3d), and the forewing costa was supported over the upward-curved lateral part of the operculum (Fig. 3e). As the insects began to produce sounds, they raised their abdomen, and in this way, the distance between abdomen and substrate was increased (S1 Video). Then, the forewings were opened and closed rapidly. During the course of forewing movement, the basal inner margin of the forewing was firmly anchored in the wing-holding groove. When the forewings returned to their resting position, the highly sclerotized costa of forewing struck the upward-curved lateral part of the operculum, which resulted in the production of the impact sound (S1 Video).

**Fig 4:**
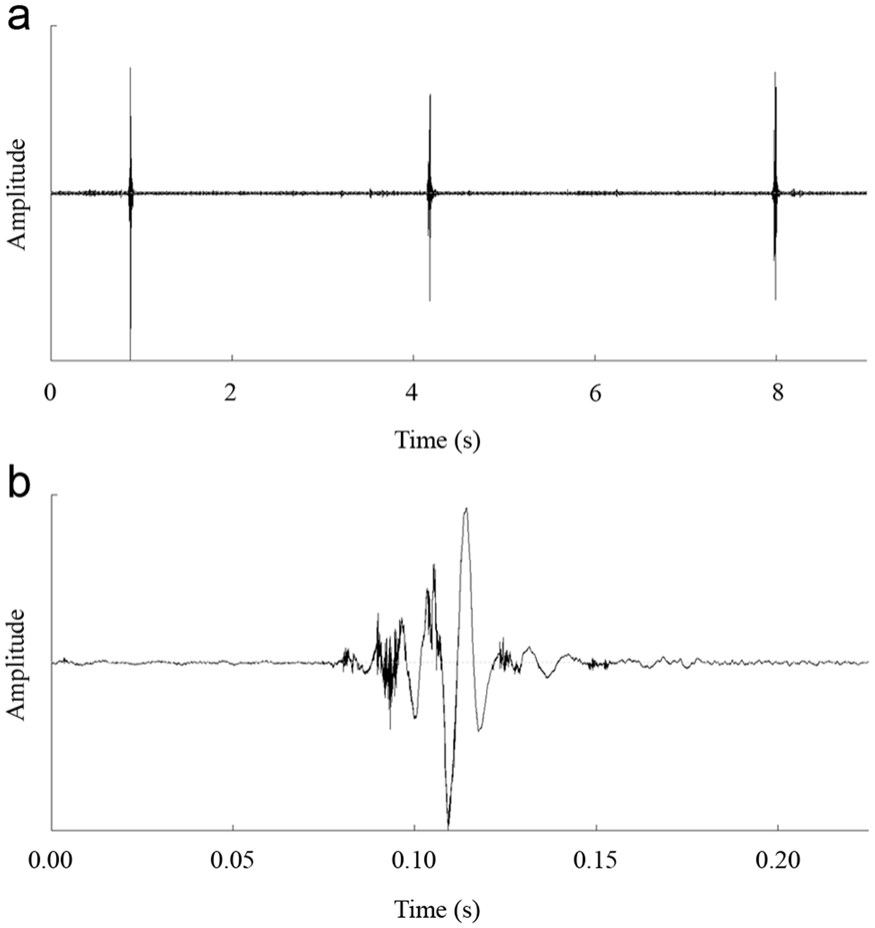
Oscillograms of sounds recorded from male *K*. *caelatata* after ablation of the costas of forewings. (a) Oscillogram of three sounds produced by a post-ablated male. (b) Detailed oscillogram of a single sound produced by a post-ablated male.

Costa-ablation experiments clearly demonstrated that the forewing costa played a critical role in sound production of *K*. *caelatata* males. When the costa of the forewing was removed, the males could normally perform the sound-producing movements (i.e., rapid opening and closing of the forewings). However, the sounds produced over the course of the movements were almost inaudible and often contaminated with background noise, which could not attract other males and females. It was difficult to record the sounds produced by the ablated males because the intensity of the sounds was very low. In order to record the sounds, the microphone was placed as close to the ablated males as possible (about 2 cm). Because the distance between the microphone and the male cicadas was very close, the sounds recorded from the ablated males might be influenced by the winds generated by movements of the forewings. Only one high-quality sound recording was obtained from an ablated male, and oscillograms of the sounds recorded from the ablated male were shown in Fig. 4. In all the 30 costa-ablation experiments, we obtained consistent results.

### Forewing and body measurements

**Fig 5:**
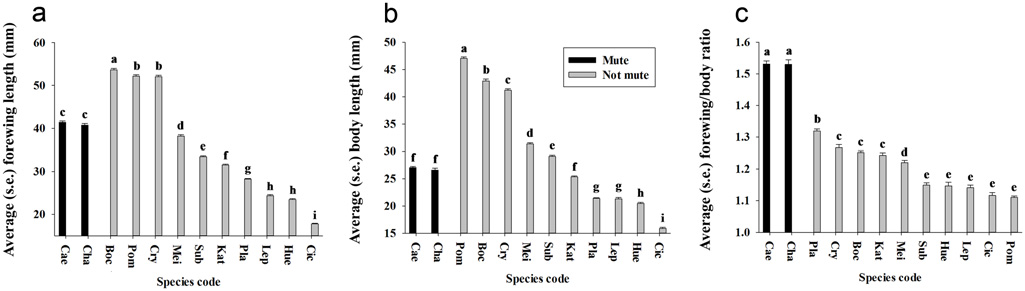
Forewing and body length of the 12 species included in this study. (a) Forewing length. (b) Body length. (c) Forewing/body ratio. Different letters represent significant difference at the 0.05-level.

We showed that the average forewing length varied significantly among most of the 12 species (Fig. 5a) (df = 283,11, F = 1893.92, *P* < 0.001), and we found that the forewing length of the two mute cicada species (*K*. *caelatata* and *K*. *chama*) were within the range of other examined cicada species. We also found that the average body length varied significantly among most of the 12 species (Fig. 5b) (df = 283,11, F = 1742.47, *P* < 0.001), and that the body length of two mute cicada species was within the range of other examined cicada species. In males of mute cicadas, a unique morphological feature is that the ratio of forewing length to body length (i.e., relative forewing length) is significantly higher than in other cicada species (df = 283,11, F = 240.07, *P* < 0.001) (Fig. 5c).

### Reflectance-based analysis of forewing costa of *K*. *caelatata* and 11 other species

Visual inspection of regular photos of forewing costa underscore that some species-specific variation is detectable by the human eye, but it would be virtually impossible to consistently separate forewing costa of *K*. *caelatata* from those of other species with a high level of accuracy (Fig. 2a). In other words, visual inspection of forewing costa cannot be considered a reliable method for identification of cicada species. Average reflectance profiles of forewing costa revealed (Fig. 2b): 1) considerable species-specific variation across the examined spectrum, 2) that the average reflectance profiles from the two mute cicada species were similar across the examined spectrum, and 3) that in different portions of the examined spectrum there appeared to be considerable separation (difference) of relative reflectance values among cicada species. Forward linear discriminant analysis was used to identify 23 spectral bands with the highest contribution to the classification of non-mute and mute cicada species, and these spectral bands were located across the examined spectrum (Fig. 2b). We used the 10 spectral bands in the training data sets to develop discriminant functions, and five separate classifications were conducted, in which 24 of the 120 reflectance profiles were randomly selected for independent validation. From this comprehensive validation exercise, we quantified both classification sensitivity (ability to positively detect mute cicadas) and specificity (ability to positively detect non-mute cicadas), and they were 100% and 97%, respectively (Table 2).

**Table 2:** Reflectance-based classification of independent validation data acquired from forewing costa.

Assigned class		
Actual class	Mute cicada	Non-mute cicada
Mute cicada	21 (100%)	0 (0%)
Non-mute cicada	3 (3%)	96 (97%)

Linear discriminant classification of 120 randomly selected reflectance profiles acquired from forewing costa was based on reflectance values in 10 spectral bands (Fig. 2b).

### Acoustic properties of the sounds produced by *Karenia caelatata* males

**Fig 6:**
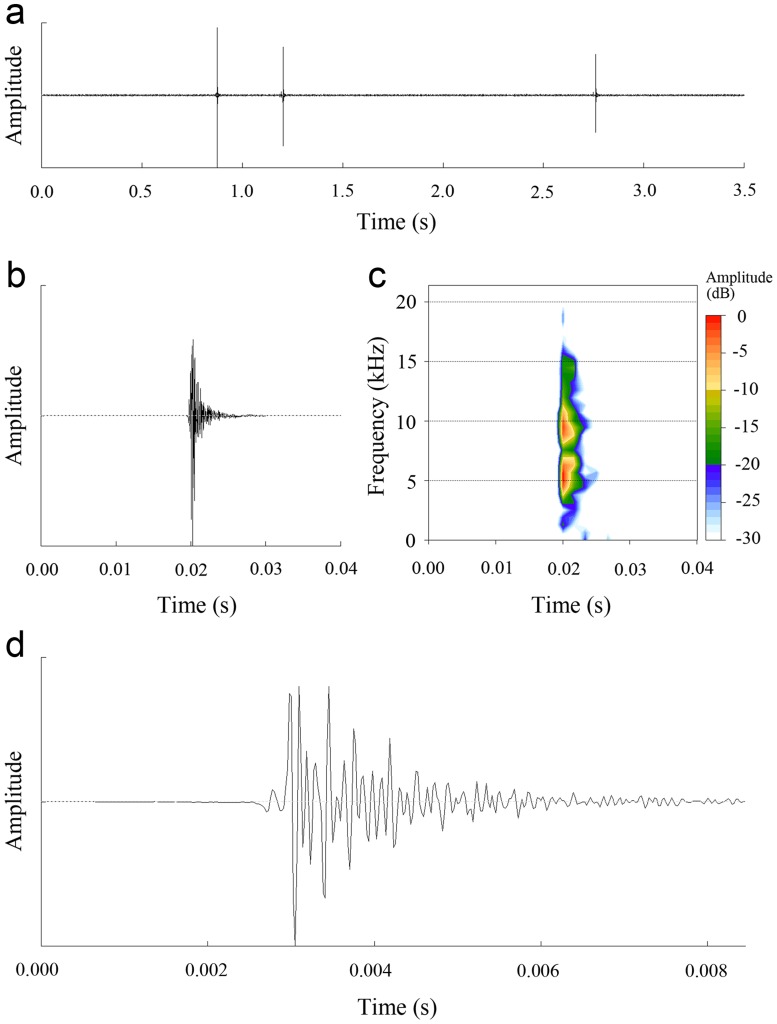
Analysis of sounds produced by male *K*. *caelatata*. (a) Oscillogram of three impact sounds produced by a male. (b, c) Oscillogram and spectrogram of an impact sound. (d) Detailed oscillogram of an impact sound.

Each opening and closing movement of the forewings was responsible for generating a single pulse (i.e., an impact sound), and a sequence of sounds were generated by multiple movements of the forewings (S1 Video; Fig. 6a, b, d). The mean interval between impact sounds was 3.36 ± 1.92 s (mean ± S.D.; range = 0.321–5.952 s; *N* = 37 from 14 males). The mean duration of the impact sound was 7.33 ± 2.32 ms (mean ± S.D.; range = 4–14 ms; *N* = 81 from 25 males). The impact sound exhibited amplitude modulation, typically showing a gradual decrease in amplitude from beginning to end (Fig. 6b, d). In the frequency domain, the impact sound contained a broad spectrum of frequencies, from 3 to 15 kHz (Fig. 6c). The main energy of the sound was concentrated in two frequency bands, from 4 to 7 kHz, and from 8 to 10 kHz (Fig. 6c). The average peaks of the first and second frequency bands were 5.77 ± 0.55 kHz (range = 5.07–6.67 kHz, *N* = 20 from 13 males), and 8.97 ± 0.67 kHz (range = 8.04–9.99 kHz, *N* = 20 from 13 males), respectively. The sound pressure level of the impact sound ranged between 61.9 and 71.0 dB SPL, measured at 0.5 m from the cicada (*N* = 25 from 17 males).

### Acoustic playback experiments

During the control period in which no sound was played back, no males and females of *K*. *caelatata* were attracted to the loudspeaker. When the sounds recorded from a *K*. *caelatata* male were broadcast in the field, male adults responded to the playbacks by flying towards the loudspeaker. The males were able to locate the sound source during flight and approached to the loudspeaker by flying from one tree to the next. The attracted cicadas landed close to the loudspeaker and walked around it, and some of them even perched on the loudspeaker. The number of males attracted to the loudspeaker in each of the 15 acoustic playback tests varied from three to eleven, with a mean value of 8 ± 1.85 (± S.D.). During the playback tests, no female cicadas were attracted to the loudspeaker.

## Discussion

The tymbal mechanism is the most common method of sound production in cicadas. However, five genera of cicadas lack tymbal organs, i.e., *Platypedia* Uhler, *Neoplatypedia* Davis, *Karenia* Distant, *Maroboduus* Distant, and *Lamotialna* Boulard [18]. Previous studies proposed that these five cicada genera lost their tymbal organs independently [13,14]. The genus *Platypedia* contains 22 species, *Karenia* five species, while the other genera comprise two species, respectively [43]. Sound-producing behavior was previously explored in only one species of these remarkable cicadas, the wing-banger cicada *Platypedia putnami*, in which sounds were generated by slapping the wings together over the body or slapping the wings against the body surface [44]. In the present study, we studied the acoustic behavior of the cicada *K*. *caelatata*, and found that the males of this species produced sounds by banging the costa of forewing against the corresponding operculum. To our knowledge, this is the first known case of a cicada species producing sounds with this type of mechanism. This expands our knowledge on the diversity of sound-producing behavior in cicadas. We hope that our results will promote further studies of sound-producing behavior in other cicada species which have lost the ability to produce sounds using tymbal mechanism.

Interestingly, previous studies have shown that some cicada species possessing the normal tymbal mechanism of sound production can also generate sounds using their wings. For example, Dugdale and Fleming [45] suggested that some New Zealand cicadas could produce sounds by banging the costal edge of forewing against the substrate. Popov [46] found that in the cicada *Cicadetta sinuatipennis*, sounds were generated by the outward buckling of a specialized caudal margin of the forewing which was locked in the wing-holding groove on the scutellum before sound production. Gogala and Trilar [47] speculated that the sounds were produced by buckling stiff mechanically bi-stable structure in the forewing in three cicadas (*Cicadatra atra*, *C*. *persica*, and *Pagiphora annulata*), and Sueur and Aubin [48] proposed similar wing sound-producing mechanism for the cicadas belonging to the genus *Tibicina*. Both these results and the results of our acoustic study on *K*. *caelatata* indicate that cicadas have evolved various mechanisms to emit sounds using their wings.

As compared with species of other cicada genera in Cicadidae, the structures involved in the sound production of mute cicadas are morphologically modified. First, the mute cicadas develop relatively longer forewing, and reflectance-based analysis of forewing costa reveals that the average reflectance profiles from the two mute cicada species are quite different from that obtained from species belonging to other cicada genera. Second, in most cicadas, the cruciform elevation on the scutellum is typically X-shaped, but in *K*. *caelatata* the two posterior angles of the cruciform elevation are almost disappeared and the wing-holding groove is located much closer to the cruciform elevation, leading the inner margin of the forewing could be firmly anchored in the wing-holding groove during the course of sound production. Third, morphology of the operculum of *K*. *caelatata* is also extremely changed, i.e., the base of the outer margin strongly curved upward and reached far beyond the lateral margin of the body. Previous studies have revealed that the operculum of many cicada species plays a special role in modulating the amplitude of the sounds produced by tymbal activity [10,49]. The operculum of *K*. *caelatata* does not have this function. However, morphological modification in the operculum makes it one of the primary sound-producing structures in *K*. *caelatata*. Once new behavior is obtained, selection should favor morphological changes that facilitate the new activity [50,51]. We suggest that morphological changes occurring in the forewing, the cruciform elevation and the operculum are highly correlated with the evolution of sound-producing behavior of *K*. *caelatata* and its allies.

The reason why cicadas of the genus *Karenia* have shifted so radically away from the most widely used sound-producing method (i.e., the tymbal mechanism) is as yet unknown. Determining what selective advantages *Karenia* cicadas gain by adopting the unusual sound-producing method is necessary to resolve this question. For example, does the sound-producing method provide an advantage in terms of energy consumption? Does it reduce the risks imposed by acoustically-orienting parasites or predators? Testing the ecological and physiological significance of the unusual sound-producing behavior will offer interesting implications for the evolution of sound production in *Karenia*.

Specialized acoustic behavior is always a communicative phenomenon in all kinds of animals [28]. Most cicada species can emit various kinds of sounds which function in different behavioral contexts (e.g., defense against predators) [52,53]. In cicadas, the calling song is the most common acoustic signal which plays a leading role in intraspecific communication [9]. In some cicada species, the calling songs can attract conspecific males in dense aggregations where they exhibit chorus activity [33,54–57]. Our observations indicated that males of *K*. *caelatata* produce only one type of sound, and acoustic playback experiments clearly revealed that *K*. *caelatata* males could be attracted by this type of sound. In addition, we observed that when the first male in the population began to emit sounds by clapping wings, more and more surrounding males followed (i.e., chorusing behavior). These observations and experiments, together with the phenomenon that males of *K*. *chama* can be easily attracted to sounds produced by clapping of hands or knocking of bamboo sticks in a rhythm similar to that produced by the insects [18], suggest that the sounds produced by males of the “mute” cicadas of the genus *Karenia* serve as calling song. In cicadas, another function of calling song is to attract conspecific females at long range or evoke acoustic responses from conspecific females [48,58–60]. In *K*. *caelatata*, playback experiments demonstrated that females did not show positive phonotaxis to the sounds emitted by the males. This implies that the sounds produced by *K*. *caelatata* males, being similar to the calling song of Australian tick-tock cicadas [58] and periodical cicadas [60], are used as signals not to attract females, but to elicit female acoustic responses. In addition, the loudspeaker was placed on the ground when we conducted the acoustic playback experiments in the field, and this may influence the efficiency of the sounds produced by male cicadas in eliciting phonotactic responses from females. Therefore, future behavioral observations and acoustic playback experiments are needed to investigate the acoustic and phonotactic responses of females in detail.

## Supporting Information

S1 Photograph Still photograph from S1 Video showing an acoustic signaling male. (JPG)

S1 Video This video shows a male cicada emitting four impact sounds. (MP4)
