# Supplementary figures and images for: How Do “Mute” Cicadas Produce Their Calling Songs?
Source: PLoS One. 2015 Feb 25;10(2):e0118554. doi: 10.1371/journal.pone.0118554 (PMC4340955; doi:10.1371/journal.pone.0118554)

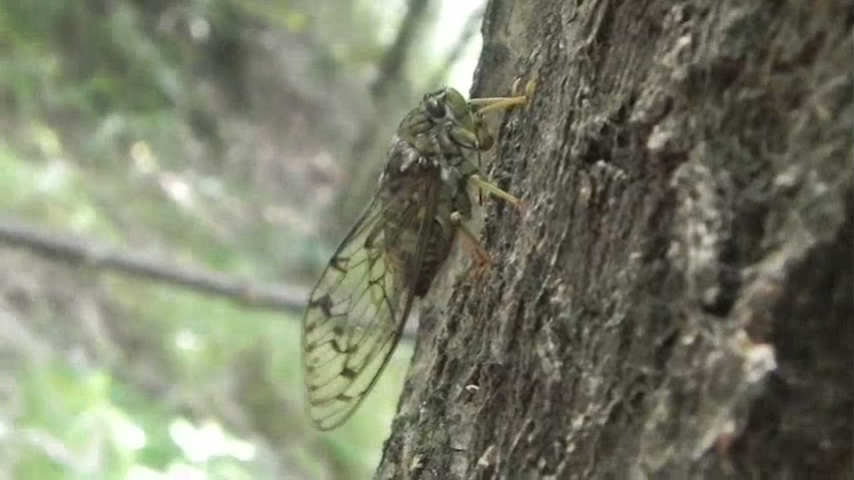

Supplement: S1 Photograph — (JPG) [file pone.0118554.s001.JPG]
